# 7-[2-(3-Fur­yl)eth­yl]-7,8-dimethyl-3,5,6,6a,7,8,9,10-octa­hydro-1*H*-naphtho­[1,8a-*c*]furan-3-one

**DOI:** 10.1107/S1600536810035373

**Published:** 2010-09-11

**Authors:** Akhtar Mohammad, Muhammad Raza Shah, Itrat Anis, Vickie McKee, Josef W. A. Frese

**Affiliations:** a Department of Chemistry, University of Karachi, Karachi 75270, Pakistan; b H.E.J. Research Institute of Chemistry, International Center for Chemical and Biological Science, University of Karachi, Karachi 75270, Pakistan; c Chemistry Department, Loughborough University, Loughborough, Leicestershire LE11 3TU, England

## Abstract

In the title mol­ecule, C_20_H_26_O_3_, a clerodane diterpenoid isolated from *Dodonaea viscosa­*, the *trans*-fused six-membered rings of the deca­line system display chair conformations. The five-membered lactone ring adopts an envelope conformation and the five-membered furan ring is essentially planar with a maximum deviation of 0.0052 (12) Å.

## Related literature

For the absolute stereochemistry of the title compound from NMR and literature data, see: Jefferies & Payne (1967 ▶). For background to natural product chemistry, see: Arfan *et al.* (2010 ▶); Khan *et al.* (2005 ▶).
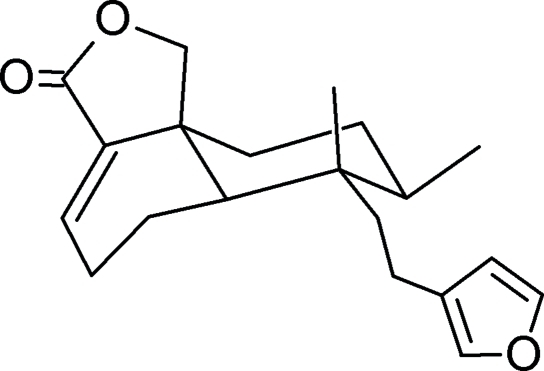


## Experimental

### 

#### Crystal data

C_20_H_26_O_3_
*M* *_r_* = 314.41
Orthorhombic, 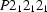

*a* = 9.1343 (8) Å
*b* = 11.8752 (10) Å
*c* = 15.5255 (13) Å
*V* = 1684.1 (2) Å^3^
*Z* = 4
Mo *K*α radiation
μ = 0.08 mm^−1^
*T* = 150 K
0.46 × 0.21 × 0.08 mm

#### Data collection

Bruker APEXII CCD diffractometer
Absorption correction: multi-scan (*SADABS*; Sheldrick, 2008*a* ▶) *T*_min_ = 0.963, *T*_max_ = 0.994
17210 measured reflections
2378 independent reflections
2124 reflections with *I* > 2σ(*I*)
*R*_int_ = 0.036

#### Refinement

*R*[*F* ^2^ > 2σ(*F* ^2^)] = 0.035
*wR*(*F* ^2^) = 0.094
*S* = 1.05
2378 reflections
210 parameters
H-atom parameters constrained
Δρ_max_ = 0.28 e Å^−3^
Δρ_min_ = −0.15 e Å^−3^

### 

Data collection: *APEX2* (Bruker, 1998 ▶); cell refinement: *SAINT* (Bruker, 1998 ▶); data reduction: *SAINT*; program(s) used to solve structure: *SHELXS97* (Sheldrick, 2008*b* ▶) and *OLEX2* (Dolomanov *et al.*, 2009 ▶); program(s) used to refine structure: *SHELXL97* (Sheldrick, 2008*b* ▶) and *OLEX2*; molecular graphics: *SHELXTL* (Sheldrick, 2008*b* ▶); software used to prepare material for publication: *SHELXTL*.

## Supplementary Material

Crystal structure: contains datablocks global, I. DOI: 10.1107/S1600536810035373/pv2325sup1.cif

Structure factors: contains datablocks I. DOI: 10.1107/S1600536810035373/pv2325Isup2.hkl

Additional supplementary materials:  crystallographic information; 3D view; checkCIF report

## Figures and Tables

**Fig. 1.**
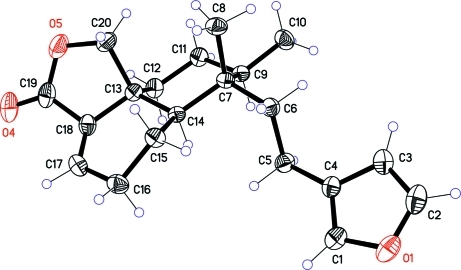
Molecular structure of the title molecule with atom labels drawn with displacement ellipsoids at 50% probability level.

**Fig. 2.**
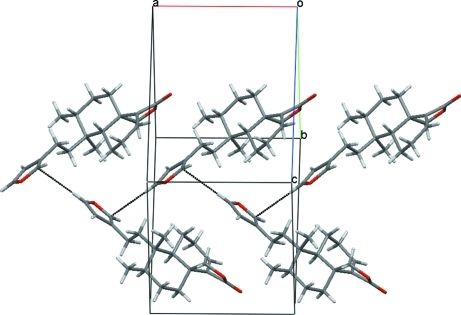
Unit cell packinig of the title compound showing C—H···π interactions as dashed lines.

## supplementary crystallographic information

### Comment

In continuation of our work on Natural Product chemistry (Arfan *et al.*, 2010) and in view of the important role played by natural products in medicinal chemistry (Khan *et al.*, 2005), the plant *Dodonaea viscosa* has been subjected to phytochemical investigation which resulted in the isolation of the title compound.

The title molecule (Fig. 1) is chiral, but its absolute configuration could not be determined from the crystallographic data. However, the absolute stereochemistry of the compound was established from NMR and literature data (Jefferies *et al.*, 1967). The molecule has four chiral centers and the two *trans* fused cyclohexyl rings of decaline, C7–C14 and C13–C18, adopt chair conformations. The five-membered lactone ring adopts a C13-envelope conformation with C13 0.625 (3) Å out of the plane formed by the rest of the ring atoms. The five-membered furan ring is essentially planar with maximum deviation of any atom from the plane being 0.0052 (12) Å for C2. There are no significant hydrogen bonds or π-π interactions between the molecules although there may be a weak C—H···π interaction linking neighbouring molecules; C2—H2···C1 3.747 (3)Å (under 1/2 + *x*, 1.5 - *y*, 1 - *z*). This leads to zigzag chains running parallel to the *a* axis (Fig. 2).

### Experimental

The whole plant of *Dodonaea viscosa* (50 kg) was powdered and extracted with methanol (100 L × 3) at room temperature and the residue (1 kg) was separated under vacuum. The residue was suspended in water and extracted with n-hexane, chloroform, ethyl acetate and n-butanol, respectively. The chloroform fraction (500 g) was subjected repeatedly to column chromatography on silica gel using petroleum ether with a gradient of 15% ethyl acetate to yield the title compound (2 g). Colourless crystals suitable for X-ray crystallographic analysis were obtained from an ether–chloroform mixture(1:1) by slow evaporation of the solvent at room temperature.

### Refinement

H atoms were placed in geometric positions using a riding model with C—H distances constrained as 0.95, 0.98 and 0.99 Å for aryl, methyl and methylene groups, respectively, and *U*_iso_(H) = 1.5*U*_eq_(C) for methyl groups and *U*_iso_(H) = 1.2*U*_eq_(C) for all others. Since the compound contains no heavy atoms an absolute configuration could not be determined; the Friedel pairs (1792) were merged.

### Crystal data

**Table:**

C_20_H_26_O_3_	*F*(000) = 680
*M**_r_* = 314.41	*D*_x_ = 1.240 Mg m^−^^3^
Orthorhombic, *P*2_1_2_1_2_1_	Mo *K*α radiation, λ = 0.71073 Å
Hall symbol: P 2ac 2ab	Cell parameters from 4592 reflections
*a* = 9.1343 (8) Å	θ = 2.6–25.8°
*b* = 11.8752 (10) Å	µ = 0.08 mm^−^^1^
*c* = 15.5255 (13) Å	*T* = 150 K
*V* = 1684.1 (2) Å^3^	Plate, colourless
*Z* = 4	0.46 × 0.21 × 0.08 mm

### Data collection

**Table:**

Bruker APEXII CCD diffractometer	2378 independent reflections
Radiation source: fine-focus sealed tube	2124 reflections with *I* > 2σ(*I*)
graphite	*R*_int_ = 0.036
ω rotation with narrow frames scans	θ_max_ = 28.3°, θ_min_ = 2.2°
Absorption correction: multi-scan (*SADABS*; Sheldrick, 2008*a*)	*h* = −12→12
*T*_min_ = 0.963, *T*_max_ = 0.994	*k* = −15→15
17210 measured reflections	*l* = −20→20

### Refinement

**Table:**

Refinement on *F*^2^	Primary atom site location: structure-invariant direct methods
Least-squares matrix: full	Secondary atom site location: difference Fourier map
*R*[*F*^2^ > 2σ(*F*^2^)] = 0.035	Hydrogen site location: inferred from neighbouring sites
*wR*(*F*^2^) = 0.094	H-atom parameters constrained
*S* = 1.05	*w* = 1/[σ^2^(*F*_o_^2^) + (0.0583*P*)^2^ + 0.1493*P*] where *P* = (*F*_o_^2^ + 2*F*_c_^2^)/3
2378 reflections	(Δ/σ)_max_ < 0.001
210 parameters	Δρ_max_ = 0.28 e Å^−^^3^
0 restraints	Δρ_min_ = −0.15 e Å^−^^3^

### Special details

**Table:**

Geometry. All e.s.d.'s (except the e.s.d. in the dihedral angle between two l.s. planes) are estimated using the full covariance matrix. The cell e.s.d.'s are taken into account individually in the estimation of e.s.d.'s in distances, angles and torsion angles; correlations between e.s.d.'s in cell parameters are only used when they are defined by crystal symmetry. An approximate (isotropic) treatment of cell e.s.d.'s is used for estimating e.s.d.'s involving l.s. planes.
Refinement. Refinement of *F*^2^ against ALL reflections. The weighted *R*-factor *wR* and goodness of fit *S* are based on *F*^2^, conventional *R*-factors *R* are based on *F*, with *F* set to zero for negative *F*^2^. The threshold expression of *F*^2^ > σ(*F*^2^) is used only for calculating *R*-factors(gt) *etc*. and is not relevant to the choice of reflections for refinement. *R*-factors based on *F*^2^ are statistically about twice as large as those based on *F*, and *R*- factors based on ALL data will be even larger.

### Fractional atomic coordinates and isotropic or equivalent isotropic displacement parameters (Å^2^)

**Table:**

	*x*	*y*	*z*	*U*_iso_*/*U*_eq_	
C1	0.7909 (2)	0.60429 (16)	0.47103 (12)	0.0302 (4)	
H1	0.7308	0.5466	0.4945	0.036*	
O1	0.90632 (16)	0.65328 (12)	0.51339 (8)	0.0364 (3)	
C2	0.9617 (2)	0.73164 (17)	0.45834 (13)	0.0363 (4)	
H2	1.0422	0.7795	0.4710	0.044*	
C3	0.8874 (2)	0.73222 (16)	0.38386 (13)	0.0313 (4)	
H3	0.9060	0.7787	0.3353	0.038*	
C4	0.77393 (19)	0.64889 (14)	0.39168 (11)	0.0243 (3)	
C5	0.6611 (2)	0.61806 (14)	0.32528 (11)	0.0263 (4)	
H5A	0.5867	0.5685	0.3520	0.032*	
H5B	0.7089	0.5754	0.2783	0.032*	
C6	0.58507 (19)	0.72228 (13)	0.28730 (11)	0.0232 (3)	
H6A	0.6615	0.7716	0.2624	0.028*	
H6B	0.5391	0.7642	0.3353	0.028*	
C7	0.46681 (18)	0.70275 (13)	0.21744 (10)	0.0205 (3)	
C8	0.4031 (2)	0.82001 (13)	0.19732 (12)	0.0274 (4)	
H8A	0.3394	0.8439	0.2447	0.041*	
H8B	0.3461	0.8165	0.1439	0.041*	
H8C	0.4831	0.8742	0.1905	0.041*	
C9	0.53645 (18)	0.64730 (14)	0.13577 (10)	0.0223 (3)	
H9	0.5889	0.5781	0.1557	0.027*	
C10	0.6498 (2)	0.72123 (16)	0.09011 (12)	0.0310 (4)	
H10A	0.6954	0.6784	0.0432	0.047*	
H10B	0.7252	0.7444	0.1314	0.047*	
H10C	0.6015	0.7881	0.0665	0.047*	
C11	0.42124 (19)	0.60881 (15)	0.06978 (10)	0.0260 (4)	
H11A	0.3736	0.6761	0.0447	0.031*	
H11B	0.4713	0.5683	0.0225	0.031*	
C12	0.30336 (19)	0.53222 (15)	0.10773 (10)	0.0256 (4)	
H12A	0.3482	0.4606	0.1268	0.031*	
H12B	0.2295	0.5147	0.0630	0.031*	
C13	0.22816 (18)	0.59026 (14)	0.18497 (10)	0.0219 (3)	
C14	0.34897 (18)	0.61938 (13)	0.25089 (10)	0.0194 (3)	
H14	0.4034	0.5471	0.2589	0.023*	
C15	0.2826 (2)	0.64366 (14)	0.33993 (10)	0.0237 (3)	
H15A	0.3606	0.6675	0.3803	0.028*	
H15B	0.2101	0.7053	0.3355	0.028*	
C16	0.2082 (2)	0.53662 (15)	0.37365 (11)	0.0280 (4)	
H16A	0.2832	0.4784	0.3853	0.034*	
H16B	0.1571	0.5535	0.4284	0.034*	
C17	0.1000 (2)	0.49250 (14)	0.30904 (12)	0.0281 (4)	
H17	0.0218	0.4459	0.3279	0.034*	
C18	0.11187 (19)	0.51761 (15)	0.22587 (11)	0.0257 (4)	
C19	−0.0169 (2)	0.52032 (18)	0.16805 (12)	0.0330 (4)	
O4	−0.11876 (15)	0.45656 (14)	0.15980 (10)	0.0450 (4)	
O5	−0.00711 (14)	0.61773 (13)	0.12226 (9)	0.0390 (3)	
C20	0.12289 (19)	0.68107 (16)	0.14996 (12)	0.0298 (4)	
H20A	0.0974	0.7361	0.1955	0.036*	
H20B	0.1674	0.7218	0.1009	0.036*	

### Atomic displacement parameters (Å^2^)

**Table:**

	*U*^11^	*U*^22^	*U*^33^	*U*^12^	*U*^13^	*U*^23^
C1	0.0278 (9)	0.0339 (9)	0.0288 (9)	0.0042 (8)	−0.0020 (7)	−0.0021 (7)
O1	0.0330 (7)	0.0472 (8)	0.0288 (6)	0.0075 (7)	−0.0079 (6)	−0.0087 (6)
C2	0.0273 (9)	0.0389 (10)	0.0426 (11)	0.0006 (8)	−0.0051 (8)	−0.0131 (9)
C3	0.0259 (8)	0.0338 (9)	0.0341 (9)	−0.0024 (8)	0.0017 (8)	−0.0050 (8)
C4	0.0216 (8)	0.0248 (8)	0.0265 (8)	0.0019 (7)	0.0001 (7)	−0.0044 (7)
C5	0.0281 (8)	0.0231 (8)	0.0277 (8)	−0.0004 (7)	−0.0053 (7)	−0.0019 (7)
C6	0.0247 (8)	0.0202 (7)	0.0245 (8)	−0.0023 (7)	−0.0018 (7)	−0.0015 (6)
C7	0.0235 (7)	0.0183 (7)	0.0196 (7)	0.0008 (6)	0.0006 (6)	0.0005 (6)
C8	0.0317 (9)	0.0192 (7)	0.0313 (9)	0.0031 (7)	0.0000 (8)	0.0022 (7)
C9	0.0239 (8)	0.0222 (7)	0.0209 (7)	0.0020 (7)	0.0008 (6)	0.0015 (6)
C10	0.0299 (9)	0.0357 (9)	0.0274 (9)	−0.0036 (8)	0.0068 (7)	0.0036 (8)
C11	0.0263 (8)	0.0331 (9)	0.0188 (7)	0.0008 (8)	0.0014 (7)	−0.0020 (7)
C12	0.0259 (8)	0.0310 (9)	0.0200 (7)	−0.0013 (7)	−0.0004 (7)	−0.0056 (7)
C13	0.0225 (8)	0.0236 (8)	0.0195 (7)	0.0014 (6)	−0.0005 (6)	−0.0016 (6)
C14	0.0208 (7)	0.0192 (7)	0.0180 (7)	0.0013 (6)	−0.0001 (6)	0.0004 (6)
C15	0.0278 (8)	0.0243 (8)	0.0190 (7)	−0.0015 (7)	0.0001 (7)	−0.0021 (6)
C16	0.0318 (9)	0.0304 (9)	0.0217 (8)	−0.0001 (8)	0.0036 (7)	0.0027 (7)
C17	0.0274 (8)	0.0258 (8)	0.0311 (8)	−0.0045 (7)	0.0064 (7)	−0.0014 (7)
C18	0.0219 (8)	0.0259 (8)	0.0293 (8)	−0.0015 (7)	0.0007 (7)	−0.0058 (7)
C19	0.0222 (8)	0.0461 (11)	0.0307 (9)	0.0012 (8)	0.0014 (8)	−0.0100 (8)
O4	0.0257 (7)	0.0606 (10)	0.0486 (8)	−0.0090 (7)	−0.0011 (7)	−0.0152 (8)
O5	0.0255 (7)	0.0542 (9)	0.0372 (7)	0.0028 (6)	−0.0079 (6)	0.0003 (7)
C20	0.0261 (9)	0.0344 (9)	0.0289 (9)	0.0061 (7)	−0.0036 (7)	0.0029 (7)

### Geometric parameters (Å, °)

**Table:**

C1—C4	1.350 (3)	C10—H10C	0.9800
C1—O1	1.372 (2)	C11—C12	1.528 (2)
C1—H1	0.9500	C11—H11A	0.9900
O1—C2	1.361 (3)	C11—H11B	0.9900
C2—C3	1.341 (3)	C12—C13	1.544 (2)
C2—H2	0.9500	C12—H12A	0.9900
C3—C4	1.438 (2)	C12—H12B	0.9900
C3—H3	0.9500	C13—C18	1.509 (2)
C4—C5	1.503 (2)	C13—C20	1.544 (2)
C5—C6	1.537 (2)	C13—C14	1.544 (2)
C5—H5A	0.9900	C14—C15	1.537 (2)
C5—H5B	0.9900	C14—H14	1.0000
C6—C7	1.548 (2)	C15—C16	1.534 (2)
C6—H6A	0.9900	C15—H15A	0.9900
C6—H6B	0.9900	C15—H15B	0.9900
C7—C8	1.541 (2)	C16—C17	1.502 (3)
C7—C14	1.552 (2)	C16—H16A	0.9900
C7—C9	1.564 (2)	C16—H16B	0.9900
C8—H8A	0.9800	C17—C18	1.330 (3)
C8—H8B	0.9800	C17—H17	0.9500
C8—H8C	0.9800	C18—C19	1.480 (2)
C9—C10	1.532 (2)	C19—O4	1.207 (2)
C9—C11	1.538 (2)	C19—O5	1.361 (3)
C9—H9	1.0000	O5—C20	1.470 (2)
C10—H10A	0.9800	C20—H20A	0.9900
C10—H10B	0.9800	C20—H20B	0.9900
			
C4—C1—O1	111.05 (17)	C9—C11—H11A	108.8
C4—C1—H1	124.5	C12—C11—H11B	108.8
O1—C1—H1	124.5	C9—C11—H11B	108.8
C2—O1—C1	105.92 (15)	H11A—C11—H11B	107.7
C3—C2—O1	110.93 (18)	C11—C12—C13	110.32 (14)
C3—C2—H2	124.5	C11—C12—H12A	109.6
O1—C2—H2	124.5	C13—C12—H12A	109.6
C2—C3—C4	106.76 (18)	C11—C12—H12B	109.6
C2—C3—H3	126.6	C13—C12—H12B	109.6
C4—C3—H3	126.6	H12A—C12—H12B	108.1
C1—C4—C3	105.33 (16)	C18—C13—C20	96.26 (13)
C1—C4—C5	127.53 (17)	C18—C13—C14	110.63 (13)
C3—C4—C5	127.14 (16)	C20—C13—C14	121.47 (14)
C4—C5—C6	112.14 (14)	C18—C13—C12	112.63 (14)
C4—C5—H5A	109.2	C20—C13—C12	108.38 (14)
C6—C5—H5A	109.2	C14—C13—C12	107.26 (13)
C4—C5—H5B	109.2	C15—C14—C13	110.88 (13)
C6—C5—H5B	109.2	C15—C14—C7	117.05 (13)
H5A—C5—H5B	107.9	C13—C14—C7	114.63 (13)
C5—C6—C7	117.61 (13)	C15—C14—H14	104.2
C5—C6—H6A	107.9	C13—C14—H14	104.2
C7—C6—H6A	107.9	C7—C14—H14	104.2
C5—C6—H6B	107.9	C16—C15—C14	109.05 (13)
C7—C6—H6B	107.9	C16—C15—H15A	109.9
H6A—C6—H6B	107.2	C14—C15—H15A	109.9
C8—C7—C6	105.68 (13)	C16—C15—H15B	109.9
C8—C7—C14	112.47 (13)	C14—C15—H15B	109.9
C6—C7—C14	110.19 (12)	H15A—C15—H15B	108.3
C8—C7—C9	111.70 (13)	C17—C16—C15	110.66 (14)
C6—C7—C9	110.32 (13)	C17—C16—H16A	109.5
C14—C7—C9	106.55 (12)	C15—C16—H16A	109.5
C7—C8—H8A	109.5	C17—C16—H16B	109.5
C7—C8—H8B	109.5	C15—C16—H16B	109.5
H8A—C8—H8B	109.5	H16A—C16—H16B	108.1
C7—C8—H8C	109.5	C18—C17—C16	121.11 (16)
H8A—C8—H8C	109.5	C18—C17—H17	119.4
H8B—C8—H8C	109.5	C16—C17—H17	119.4
C10—C9—C11	108.94 (14)	C17—C18—C19	121.95 (17)
C10—C9—C7	114.14 (14)	C17—C18—C13	126.47 (16)
C11—C9—C7	112.76 (13)	C19—C18—C13	106.96 (15)
C10—C9—H9	106.9	O4—C19—O5	121.90 (18)
C11—C9—H9	106.9	O4—C19—C18	131.6 (2)
C7—C9—H9	106.9	O5—C19—C18	106.47 (15)
C9—C10—H10A	109.5	C19—O5—C20	109.57 (14)
C9—C10—H10B	109.5	O5—C20—C13	104.40 (14)
H10A—C10—H10B	109.5	O5—C20—H20A	110.9
C9—C10—H10C	109.5	C13—C20—H20A	110.9
H10A—C10—H10C	109.5	O5—C20—H20B	110.9
H10B—C10—H10C	109.5	C13—C20—H20B	110.9
C12—C11—C9	113.72 (13)	H20A—C20—H20B	108.9
C12—C11—H11A	108.8		
			
C4—C1—O1—C2	0.5 (2)	C12—C13—C14—C7	62.01 (17)
C1—O1—C2—C3	−0.8 (2)	C8—C7—C14—C15	−67.88 (18)
O1—C2—C3—C4	0.8 (2)	C6—C7—C14—C15	49.74 (18)
O1—C1—C4—C3	−0.1 (2)	C9—C7—C14—C15	169.42 (13)
O1—C1—C4—C5	179.74 (15)	C8—C7—C14—C13	64.53 (18)
C2—C3—C4—C1	−0.4 (2)	C6—C7—C14—C13	−177.85 (13)
C2—C3—C4—C5	179.75 (17)	C9—C7—C14—C13	−58.16 (16)
C1—C4—C5—C6	131.66 (19)	C13—C14—C15—C16	63.59 (17)
C3—C4—C5—C6	−48.6 (2)	C7—C14—C15—C16	−162.32 (14)
C4—C5—C6—C7	179.56 (14)	C14—C15—C16—C17	−53.83 (19)
C5—C6—C7—C8	175.77 (15)	C15—C16—C17—C18	23.3 (2)
C5—C6—C7—C14	54.02 (18)	C16—C17—C18—C19	−152.89 (18)
C5—C6—C7—C9	−63.35 (18)	C16—C17—C18—C13	−0.2 (3)
C8—C7—C9—C10	53.24 (19)	C20—C13—C18—C17	−118.6 (2)
C6—C7—C9—C10	−63.98 (17)	C14—C13—C18—C17	8.4 (3)
C14—C7—C9—C10	176.42 (14)	C12—C13—C18—C17	128.46 (19)
C8—C7—C9—C11	−71.78 (18)	C20—C13—C18—C19	37.37 (17)
C6—C7—C9—C11	171.00 (13)	C14—C13—C18—C19	164.41 (14)
C14—C7—C9—C11	51.41 (16)	C12—C13—C18—C19	−75.55 (18)
C10—C9—C11—C12	178.88 (14)	C17—C18—C19—O4	−44.6 (3)
C7—C9—C11—C12	−53.32 (19)	C13—C18—C19—O4	158.06 (19)
C9—C11—C12—C13	55.58 (19)	C17—C18—C19—O5	132.85 (18)
C11—C12—C13—C18	−179.20 (14)	C13—C18—C19—O5	−24.47 (19)
C11—C12—C13—C20	75.54 (17)	O4—C19—O5—C20	176.28 (17)
C11—C12—C13—C14	−57.24 (17)	C18—C19—O5—C20	−1.49 (19)
C18—C13—C14—C15	−39.51 (18)	C19—O5—C20—C13	25.98 (18)
C20—C13—C14—C15	72.02 (19)	C18—C13—C20—O5	−37.35 (15)
C12—C13—C14—C15	−162.72 (13)	C14—C13—C20—O5	−156.21 (14)
C18—C13—C14—C7	−174.78 (13)	C12—C13—C20—O5	79.04 (16)
C20—C13—C14—C7	−63.25 (19)		
